# James William White

**Published:** 1891-06

**Authors:** Levi Teal, A. W. Carey

**Affiliations:** Chairman.; Secretary.


### Obituary.

DIED, at Philadelphia, Pa., May 27, 1891, of heart-failure, JAMES WILLIAM WHITE, M. D., D. D. S., A. M., in the sixty-fifth year of his age.

A brief notice, in the June number of the *Dental Cosmos* just after its issue from the press, announced the death of its editor, Dr. James W. White.

Death came suddenly, unexpectedly. The previous day Dr. White was at the office as usual. He referred several times to an uncomfortable sensation in the region of the heart, but pursued his duties with his accustomed cheerfulness and diligence, and was the last to leave the building. According to his wont he spent the evening with his family in social converse, and retired without premonition that within a few short hours his eyes would close forever. He awoke unusually early, and spoke to his wife of feeling "dyspeptic," but shortly afterward announced that he was better, and would dress for the day. While so engaged he fell forward, dying almost instantly.

Last winter he had a severe attack of a somewhat obscure character, with symptoms resembling those of rheumatic pharyngitis. This had annoyed him at intervals for many years, but the last attack was the most protracted, and he recovered from it slowly, though its effects were gradually wearing away, and his friends and business associates were looking forward to a long continuance of his useful and honorable career. But it was not to be so, His work is done; he has entered into the eternal rest. Those who mourn him have the sad comfort of knowing that, however the suddenness of his taking away may have shocked them, he died as he wished to die, in the full vigor of his ripened powers. No man had a deeper horror of living to a decrepit old age, to become a care to those his life was spent in caring for. He leaves a widow, and three sons, Professor J. Williams White, of the University of Pennsylvania; Samuel Stockton White, and Louis Piers White.

The funeral services were conducted at the residence, 2012 Green street, on Friday, the 29th of May, by Rev. Dr. Edwin C. Sweetser, of the Universalist Church of the Messiah, assisted by Rev. Dr. William H. Furness. The interment was private, at Woodlands Cemetery. The pall-bearers were Mr. George W. Childs, Dr. D. Hayes Agnew, Mr. Horace Howard Furnes, Mr. Joseph C. Fraley, Dr. H. C. Wood, Dr. Lewis D. Harlow, Dr. George W. Ellis, Mr. Samuel T. Jones, Mr. S. R. Bingham, Dr. W. Storer How, Mr. John H. Long, and Mr. Edward H. Hance.

James W. White, the youngest child of William Rose and Mary (Stockton) White, was born September 29, 1826, at Hulmeville, Bucks County Pennsylvania. On his father's side he was descended from Henry White, who settled in James City County, Virginia, some time previous to or about 1649; and through his mother, from Richard Stockton, who came to Flushing, Long Island, from England about 1656, and one of whose descendants was Richard Stockton, the signer of the Declaration of Independence. When he was four years old his father died, and his mother removed with her children to her native place, Burlington, New Jersey. In his fifteenth year (13th May, 1841) he entered the establishment of his uncle, the late Samuel W. Stockton, of Philadelphia, whither his elder brother Samuel had preceded him, to learn the "art and mystery of the manufacture of incorruptible teeth." In 1844 the elder brother began business in the same branch of manufacture, on his own account, and James engaged with him.

In a speech reminiscent of the early days of dentistry Dr. White, referring to this period, some time since described himself as "the entire working force of the establishment," which was a garret at the corner of Seventh and Race streets, where he "ground the crude materials in a hand mortar, compounded them, and carried through all the processes to the finished products,—such as they were,—then turned travelling salesman, and exchanged them for currency."

The connection thus begun continued, with one or two brief interruptions, until his death. He saw the seed planted, watched it germinate, and helped to nurture its healthy growth to magnificent proportions. With every step of the development of the great business which grew from the humblest beginning until it overtopped all others of its kind in the world he was closely identified.

In 1879 Dr. Samuel S. White died, and in 1881 the S. S. White Dental Manufacturing Company was organized with a paid-in capital of one million dollars. The recognized executive and administrative ability of Dr. James W. White, and his intimate knowledge of the business, made him the logical head of the new company, and he was unanimously elected its president. That he well fulfilled expectation is shown by the unflagging prosperity of the house, which has steadily gone forward under his administration, and by the fact that he was honored by an undisputed re-election to the presidency of the company with each succeeding year. The business grew constantly, involving the necessity of increased facilities, enlarged factories, and a greater number of employes, each bringing additional care and labor to the president, who bore the burden easily, cheerfully and efficiently.

Besides his connection with the house with which his life-work was identified, Dr. White was at the time of his death the senior member of the well-known firm of manufacturing chemists, Hance Brothers & White, although not actively engaged in the conduct of the business, and a member of the board of directors of the German-American Title and Trust Company.

He studied medicine at the University of Pennsylvania, and received the degree of M. D. from that institution. He never, however, followed medicine as a vocation, though he practiced somewhat extensively among his relatives and acquaintances, as well as among the poor, and was frequently called in consultation by eminent practitioners because of his exceptional ability as a physician. The honorary degree of D. D. S. was conferred upon him by the Pennsylvania College of Dental Surgery, and the degree of A. M. by St. Lawrence University, of Canton, New York.

Entering early into the practical affairs of life, his opportunities for acquiring the education of the schools were limited, but with the aid of an intellect of the highest order and a phenomenal memory he became one of the best-informed men of his day. He had read extensively upon almost every topic of human interest, and a fact once within his grasp never escaped, never seemed to be lost sight of even for a moment. As a consequence, his mind was a veritable storehouse of knowledge, the case with which he drew upon it suggesting at times the thought that whatever he learned was mentally labeled, indexed and filed away in an orderly manner where it would be found when wanted. Method was a part of his nature. Whatever he did was done promptly, neatly and methodically, and therein lay the secret of his remarkable executive ability.

Although not posing as an orator, he was, on occasion, an excellent public speaker, one who charmed by his concise, expressive diction, apt illustration, and convincing logic.

His bent was distinctly literary. He was a readily, forcible, and polished writer, having a rare faculty of condensation,—of saying much in few words, of making every phrase pregnant with meaning,—and an illustrative and descriptive talent rarely equaled; qualities which, conjoined with an exhaustive knowledge of any subject upon which he wrote, gave to the productions of his pen a clearness and completeness that always commanded attention. His contributions to dental and medical literature were numerous and important. Upon such subjects as the diseases connected with dentition, and the general physiological and pathological relations of the teeth to the whole economy or to abnormal conditions of remote organs, he was recognized as the highest authority in the world. He was frequently solicited to prepare papers upon these topics, and always responded willingly.

Dentistry is indebted to him for several volumes of great practical value. Among them may be named "Dental Materia Medica," "Taking Impressions of the Mouth," and "The Mouth and the Teeth." He was the author of the exhaustive presentation of "Diseases Incident to the First Dentition," in the American System of

Dentistry; and also of a little pamphlet, "The Teeth, Natural and Artificial," which has had a circulation of hundreds of thousands, spreading everywhere among the people an accurate knowledge of the material facts about the teeth and the necessity and means of proper care for their preservation.

But it was as editor of the *Dental Cosmos* that his highest literary ability was exhibited and the deepest impress of his virile mind upon dental literature was made. Here was a field for which he was so peculiarly fitted that his work in it was marked by that quality for which the English language contains but one word,—genius. No other fitly characterizes the faculties which so exalted the aims, broadened the scope, and enlarged the achievements of the *Dental Cosmos* that its editorship should be accounted the "proudest position, the highest honor, which dentistry has to offer any man." These words were spoken since Dr. White's death by a practitioner of dentistry favorably known to the profession everywhere.

It was perhaps Dr. White's proudest boast that no number of the *Dental Cosmos*, from its establishment in 1850, had been issued without his personal supervision. His name did not appear as its responsible editor until 1872, but his broad knowledge, his exquisite literary taste, his keen insight, his rare judgment, were in its service from the first, ever guiding it onward and upward to the ideal of perfect journalism. To his mind, the journalistic literature of a scientific profession did not fulfill the object of its being if it was merely a record of passing events or a vehicle for the exchange of the common information of the day,—the iteration and reiteration of truths known to all men. It should be the prophet of the higher aspirations of the profession it represented, the torch-bearer of a wider knowledge, the teacher of a nobler science, the inspiration of a better practice. It could not stand still; the ideal of yesterday should be the actual of to-day, with a yet higher goal set for to-morrow's effort. Each issue should be a symposium of the freshest thought of the brightest minds, to the end that those who read should have a constant stimulus to excel and thus in time to offer their best fruits upon a common altar. This was the ideal which he endeavored to realize in his conduct of the *Dental Cosmos.* How well he succeeded is shown by the above-quoted remark, which expresses in no exaggerated form the sentiments of the vast majority of the profession.

The last contribution which Dr. White made to the *Dental Cosmos* was a characterization of his old friend, Professor Joseph Leidy, who had died less than a month before him. Its closing sentences so fittingly and accurately describe Dr. White himself that they are reproduced here:

"Appreciation of his rare intellectual gifts was forgotten in admiration of his sincere, sweet-tempered, loving nature. He was singularly modest, retiring and unassuming; genial and kindly in spirit and manner,—the friend of all, the enemy of none; a walking encyclopædia of information, as approachable as a child, ready at all times and with evident pleasure to give the benefit of his knowledge to all who sought it. His death will be mourned wherever science is valued throughout the earth, but Philadelphians especially will miss his kindly face, his ready hand, his cordial greeting, and his noble example of industry, integrity and manly character."

The key-note of his thought, whether in religion, literature, political or social science, or on professional subjects, was liberality, a catholic tolerance for views honestly held, however at variance with his own. Denominationally he was a Universalist, for many years moderator of the Church of the Messiah. Himself a man of strong conviction, and courageous in upholding them, with the broadest charity for the opinions of others, he insisted that every one should not merely have the right but owed the duty of thinking and speaking for himself. As secretary of the "People's Literary Institute," and chairman of its lecture committee, he managed it successfully for seven years,—

before and during the war,—and worked energetically in the maintenance of freedom of speech against much and bitter opposition, including at one time vigorous proceedings by the mayor of the city.

He was identified with the Freedman's Aid Society; an active worker in the Sanitary Fair, and, as chairman of the Committee on Orations and Lectures of that great enterprise, secured the substantial sum of ten thousand dollars toward the grand total. He was a member of the commission appointed by the University of Pennsylvania, under the will of the late Henry Seybert, to investigate the phenomena of spiritualism. He was treasurer of the McQuillen fund, of the Webb fund, and of the Dental and Oral Surgery Section of the IXth International Medical Congress.

Although always taking a keen interest in political affairs, he never held public office but once, and his official career was a wholesome example. When the present city charter went into operation in 1887, Dr. White's well-known reputation as a practical philanthropist gained him, without solicitation on his part, the appointment of President of the Board of Charities and Correction, a department of the city government having charge of the public hospitals and correctional institutions. The position was one of honor and hard work, without pay. He reorganized the department thoroughly, placing the administration of the various institutions upon a civil service basis, and, after serving with conspicuous fidelity for two years, was removed for refusing to acquiesce in a violation of both the letter and the principles of the civil service laws of the city, for the maintenance of which he was appointed. The entire intelligent and reputable press of Philadelphia sustained him in his position in this matter, regardless of party affiliations, and with a unanimity as to both his personal character and the great value of his public service which was a tribute as gratifying as it was unique.

Another example of his practical philanthropic work is the Maternity Hospital, of which he was one of the organizers and the president from its foundation in 1872 to his death. Its firm establishment and successful operation was largely due to his untiring zeal. He was also treasurer of the Siberian Exile Relief Association.

These were a few of his public benefactions. Privately he dispensed of his means in the relief of distress with a liberal hand, and labored unweariedly to better the condition of the afflicted and unfortunate.

Without a trace of sentimentality, he loved his kind, and did what he could to make the world brighter and better for his presence in it. His relations with employes were of the most friendly and cordial nature. The universal feeling toward him among those employed in the great establishment of which he was the executive head is thus expressed by one of them: "During thirty-eight years' continuous association not a word or act or look from him has cast a shadow of unpleasantness, and I doubt not the others have similar remembrance."

After all, it is what a man does that reveals his inner nature. From whatever point the view is taken, Dr. White, judged by his deeds, was a man of supreme ability, of high purpose, of noble aspiration, of untiring assiduity, —in a word, a strong, manly man. He had the moral courage to uphold what he thought was right, the physical courage to strike a sturdy blow for that right. Of the highest moral rectitude and strictest probity, he could pity the weakness or folly of those who stumbled and fell because unable to resist temptation, and help them to their feet again. He had an innate tact, a capacity to harmonize difficulties and organize success, that gave him marked administrative ability. Whatever he undertook to do was pursued intelligently, industriously and diligently to the end.

But he was, above all other things, a lovable man,—a friend to depend on in sickness or in health. He had that indescribable quality sometimes called personal magnetism, which attracted people to him; a genial humor which charmed them, and a sterling worth that bound them firmly to his side. As an entertaining conversationalist he had few equals. Besides his wide grasp of real knowledge, he possessed a fund of anecdote that was inexhaustible. Every incident reminded him of a story, in the telling of which he was inimitable. Always cheerful, always frank, always helpful, he will be missed by a wide circle of friends who will not soon forget his kindly ways. The *Public Ledger*, in an appreciative editorial reference to his death, said, " 'Benedicite fratercule' were the words which welcomed him into a little organization to which he belonged. 'Benedicite fratercule' will be echoed by many hearts which are sorrowful at parting with him."

"A blessing upon you, little brother," even as you diffused blessings when you were here!—F. L. HISE in *Dental Cosmos.*

---

The directors and officers of The S. S. White Dental Manufacturing Company, at a special meeting held June 3, 1891, unanimously adopted the following minute regarding the death of Dr. White:

The board of directors and the officers of The S. S. White Dental Manufacturing Company desire to express their sorrow for the loss that has befallen them in the very sudden and unexpected death of their associate and friend, Dr. James W. White, who has been president of this company from its organization.

He was a man of a high order of ability and wide scientific attainment; of great energy and tireless industry; of genial temperament and generous disposition; of dauntless courage, and pronounced in convictions which he was ready and able to maintain upon proper occasion in any presence; of perfect integrity and faithful to every trust.

Identified as he was with this business from its very beginning, forty-seven years ago, his long experience as a manufacturer and his great administrative and executive talent rendered him invaluable to the company.

As editor of the *Dental Cosmos* his name is a household word among dentists all over the world, and his unequaled skill and judgment in that direction have placed the journal very far in the front of dental periodical literature.

His contributions to dental and medical research have been widely quoted and generally accepted as of great value.

We mourn his loss. We shall miss his energetic work, his great experience, his mature judgment, his genial disposition, and his kindly ministrations, always at our disposal in time of need.

We direct that this minute be placed upon record, and a copy of it sent to his bereaved widow and children, accompanied by the expression of our deep sympathy with them in this great affliction.

J. M. LONGACRE, J. CLARENCE WHITE,
S. S. WHITE, JR., H. M. LEWIS,
W. H. GILBERT, S. T. JONES.
A. K. JOHNSTON.

---

At a special meeting of the stockholders of The S. S. White Dental Manufacturing Company, June 15, 1891, called to consider the death of the late president, Dr. James W. White, the following was unanimously adopted:

The stockholders of The S. S. White Dental Manufacturing Company, assembled in special meeting at the call of the board of directors, desire to record the expression of their sorrow for their loss by the death of Dr. James W. White, the president of this company from its foundation to the time of his death.

Most of us have been in almost daily contact with him for many years. Highly gifted by nature with the qualities of a wise and efficient executive, his experience of nearly half a century in the business had given him an accurate knowledge of all its details, and he was thus able to guide it into safe and profitable channels.

A man of great literary ability, both as writer and editor, he placed the *Dental Cosmos* at the head of dental periodical literature, and held it there without even an approach to rivalry.

We valued him not alone for his services to this company, but also for his character as a man; for his genial, kindly ways; his wise counsel, whether in business or private affairs; as a friend to whom we could go freely, certain of a patient hearing and faithful advice; for the unfailing generosity and benevolence of which we were constant witnesses, involving not alone his purse, but his great skill as a physician, which he placed freely at the service of any who needed it.

By his death this company has lost an able, devoted, and most valuable officer; the world has lost a good man, whose life was filled with kind acts and useful works; and we have lost a true and faithful friend.

We direct that this minute be filed among the records of the company, and that a copy of it be sent to his bereaved widow and children.

LEVI TEAL, *Chairman.*

A. W. CAREY, *Secretary.*

